# Comment on “Enhanced Charge Selectivity via Anodic-C_60_ Layer Reduces Nonradiative Losses in Organic Solar Cells”

**DOI:** 10.1021/acsami.1c05333

**Published:** 2022-02-03

**Authors:** Gert-Jan A. H. Wetzelaer, Paul W. M. Blom

**Affiliations:** Max Planck Institute for Polymer Research, Ackermannweg 10, Mainz55128, Germany

**Keywords:** organic solar cells, Ohmic contacts, charge selectivity, interfacial layers, organic light-emitting diodes

## Abstract

Understanding interface-related phenomena is important for improving the performance of thin-film solar cells. In *ACS Appl. Mater. Interfaces***2021**, 13, 12603–12609, Pranav et al. report that incorporating a thin C_60_ interlayer at the MoO_3_ anode results in reduced surface recombination of electrons, which is ascribed to a decreased electron accumulation near the anode on account of an increased built-in voltage. Here, we offer an alternative explanation: the introduction of a C_60_ interlayer renders the MoO_3_ contact Ohmic. The reduced anode barrier simultaneously increases the built-in voltage, minimizes nonradiative voltage losses upon the extraction of majority carriers (holes), and suppresses minority-carrier (electron) surface recombination, the latter being the result of hole accumulation and associated band bending near the Ohmic hole contact. We therefore argue that Ohmic contact formation suppresses both majority- and minority-carrier surface recombination losses, whereas the built-in voltage per se does not play a major role in this respect.

In organic photovoltaics, energy losses due to nonradiative recombination and the energetic offset between donor and acceptor materials are currently the main limiting factors.^1^ These factors result in voltage losses, which affect the attainable open-circuit voltage.^1,2^ In the early days of organic photovoltaics, it was soon recognized the work functions of the electrodes impact the open-circuit voltage by modifying contact barriers.^3,4^ Later, non-Ohmic contacts were also associated with nonradiative losses via surface recombination.^5,6^ Therefore, improving charge contacts is of paramount importance for the development of highly efficient organic solar cells.

In *ACS Appl. Mater. Interfaces***2021**, 13, 12603–12609, Pranav et al.^7^ demonstrate that the incorporation of a tunneling C_60_ interlayer at the anode of organic solar cells can increase their efficiency by reducing surface recombination losses. The authors ascribe the reduced surface recombination to an increased built-in voltage, which reduces the accumulation of minority carriers (electrons) near the anode. As such, minority carriers would not be lost at the anode interface, thereby decreasing voltage losses due to nonradiative surface recombination. Here, we would like to provide an alternative explanation for the reduction in surface recombination, being due to the formation of an Ohmic hole contact, which is accompanied by diffused hole accumulation and corresponding band bending near the anode interface. Furthermore, the Ohmic contact minimizes nonradiative voltage losses upon extraction of majority carriers.

Recently, we demonstrated that the insertion of a thin interlayer of several nanometers universally results in the formation of an Ohmic hole contact between high-work-function transition metal oxides, like MoO_3_, and organic semiconductors.^8^ Although such metal oxides form an energetic barrier for holes upon direct contact with an organic semiconductor because of electrostatic interactions, the interlayer acts as a spacer layer, decoupling the electrode from the organic semiconductor. The key requirement for the formation of an Ohmic hole contact is that the interlayer has a higher ionization energy than the organic semiconductor, which results in realignment of the Fermi level of the metal oxide and the highest occupied molecular orbital (HOMO) of the organic semiconductor. As C_60_ has a high ionization energy of 6.4 eV, this is an ideal candidate to be used as an interlayer to form Ohmic contacts with organic semiconductors with high ionization energies, even surpassing 6 eV.^8^Figure 1 shows a schematic energy band diagram of such an interlayer-enhanced contact.

**Figure 1:**
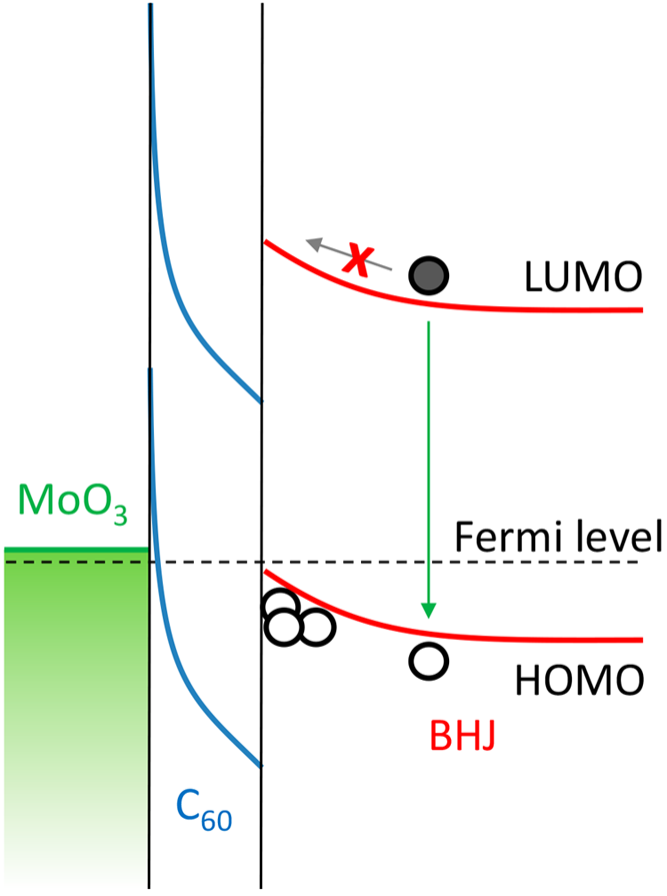
Schematic representation of the energy band diagram of an organic bulk heterojunction (BHJ) with a MoO_3_ anode and a C_60_ interlayer, based on calculations with the Poisson equation and ultraviolet photoelectron spectroscopy of such systems.^8^ The Fermi-level alignment with the HOMO of the BHJ at the interface renders the contact Ohmic, which is associated with hole accumulation and band bending. The high hole density and corresponding band bending prevents electrons from reaching the C_60_ interface (gray arrow) but allow for radiative bimolecular recombination (green arrow).

In agreement with what the authors have found, the optimum thickness for such an interlayer is 3–5 nm,^8^ which provides electrostatic decoupling on the one hand and allows charge tunneling on the other. The concept has been verified for a large range of interlayers and organic semiconductors, showing its universality. As an example, Ohmic contact formation has been demonstrated for C_60_ interlayers and triarylamine-based hole-transport materials, such as also used here by Pranav et al.^7^

The formation of an Ohmic hole contact by inserting a C_60_ interlayer provides a straightforward explanation for the increased built-in voltage, simply lowering the energy barrier (∼0.4 eV^8^) at the anode that is present when only MoO_3_ is being used. We note that an increased built-in voltage by insertion of a C_60_ interlayer has been verified experimentally for an ultraviolet-emitting organic light-emitting diode (OLED), where the reduction of the injection barrier at the anode increased the electroluminescence efficiency by several orders of magnitude.^8^ The main effect of an increased built-in voltage in a solar cell is that it allows for a higher applied voltage to partly compensate the built-in field, and thus higher open-circuit voltages are obtained.^4,6^ When expressed in terms of nonradiative voltage losses, energy barriers at the electrodes first and foremost result in voltage losses upon the extraction of majority carriers,^6^ which was not discussed by Pranav et al.^7^ Therefore, one could argue that the formation of Ohmic contacts reduces losses due to majority-carrier surface recombination.

With regard to minority-carrier surface recombination, there is an alternative and more important effect than a modified built-in voltage, which is different from the explanations the authors have provided in ref (7). Upon formation of an Ohmic hole contact, holes diffuse into the active layer to establish thermodynamic equilibrium across the interface.^8^ The accumulation of holes near the anode causes band bending. The high density of holes near the electrode prevents electrons from reaching the contact, as the sharp band bending imposes an electric field in the other direction. Therefore, nonradiative surface recombination is effectively suppressed. Instead, electrons rather recombine radiatively with the high density of free holes near the electrode (Figure 1). Indeed, Kniepert et al. have shown that for solar cells with Ohmic contacts, the influence of surface recombination is negligible when considering realistic bulk recombination,^9^ limiting nonradiative losses to a minimum.

To confirm the reduction of surface recombination by Ohmic contacts, we carried out drift-diffusion simulations^10^ of a solar cell, either with an Ohmic anode, or with a 0.4 eV barrier at the anode, corresponding to the situation with and without a C_60_ interlayer, respectively. The surface-recombination velocity is implicitly assumed to be infinite and tunneling resistance was neglected.^8^ As shown in Figure 2a, the presence of an anode barrier decreases the open-circuit voltage. Even though the hole mobility is considered to be substantially lower than the electron mobility and bimolecular recombination is reduced by a factor of 100 compared to Langevin recombination, the contribution of electrons exiting the device at the “wrong” electrode (anode) at the maximum power point is minor, even when a barrier at the anode is present, as demonstrated in Figure 2b. In the case of an Ohmic anode, minority-carrier surface recombination is completely suppressed. From these observations, it can be concluded that the voltage losses in the case of a barrier mainly originate from direct energy loss upon extraction of majority carriers (holes).

**Figure 2:**
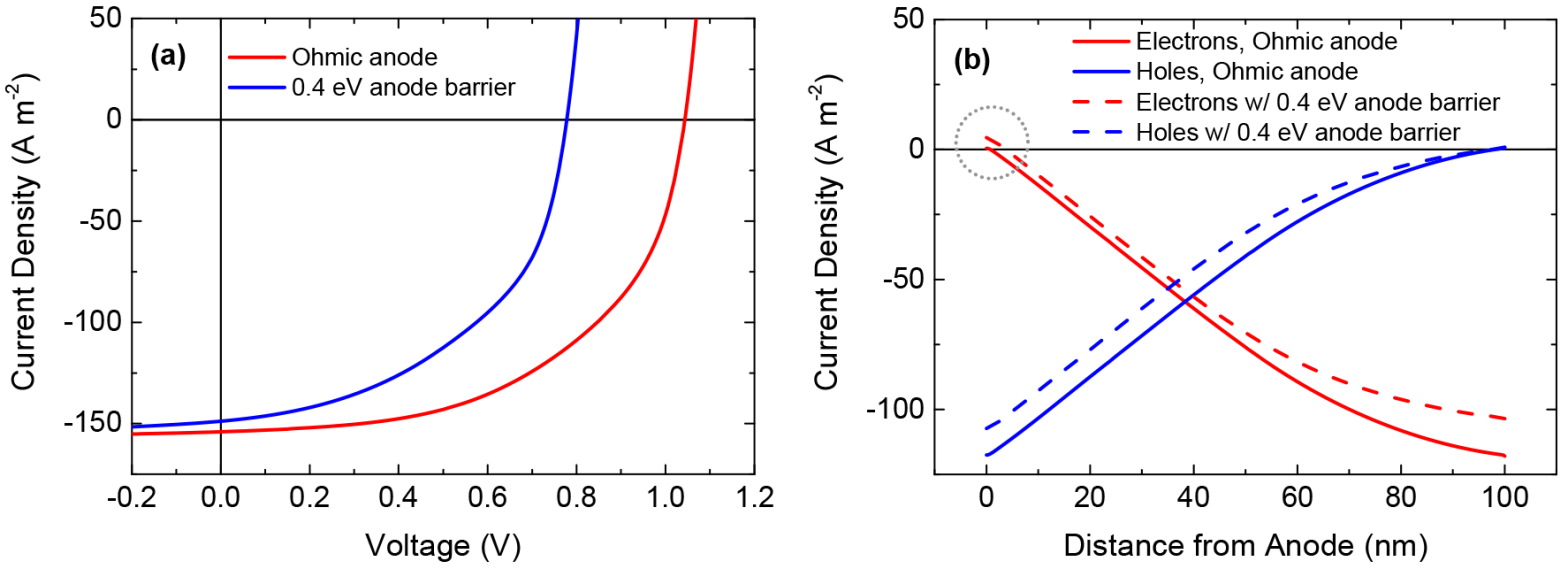
(a) Simulated current density–voltage characteristics of a solar cell with and without an anode barrier of 0.4 eV. The simulations are based on an energy gap of 1.5 eV. The mobility equals 10^–7^ m^2^ V^–1^ s^–1^ for electrons and 3 × 10^–9^ m^2^ V^–1^ s^–1^ for holes and a Langevin reduction factor of 0.01 is assumed for bimolecular recombination. (b) Current density across the photoactive layer at the maximum power point of the solar cell. A barrier at the anode results in a small contribution of electron current escaping at the anode (minority-carrier surface recombination), while an ohmic hole contact suppresses this contribution (indicated by dotted circle). The small contribution of minority-carrier surface recombination demonstrates that a barrier mainly causes voltage losses due to energy loss upon majority-carrier extraction.

We further note that in the MIS-CELIV (metal–insulator–semiconductor charge extraction by a linearly increasing voltage) measurement by Pranav et al.,^7^ hole diffusion/injection from the C_60_-improved contact may have suppressed the buildup of an electron reservoir near the TAPC electron-blocking layer, which can be emptied by bimolecular recombination. Such an effect is expected when the hole contact is rendered Ohmic, which is reached to its full extent when the C_60_ interlayer surpasses a thickness 2 nm. This provides an explanation alternative to an increase in surface-recombination velocity upon introducing a C_60_ interlayer, which would anyway be unlikely given the large barrier induced by the low electron affinity (∼2 eV^11^) of the TAPC electron-blocking layer, which would be the rate-limiting factor with regard to surface recombination of electrons.

Further experimental evidence for the charge selectivity induced by Ohmic contacts formed with the universal interlayer strategy is provided by our recent work on single-layer organic light-emitting diodes (OLEDs).^12^ By using a 3 nm C_60_ interlayer, an Ohmic hole contact was formed between MoO_3_ and the efficient emitter CzDBA. Especially in OLEDs, any nonradiative surface recombination would be detrimental for the efficiency of the device. Despite the high electron affinity and low energy gap of C_60_, which conventionally would be expected to be a sink for both electrons and excitons, the external quantum efficiency of the OLED reached almost 20%,^12^ which amounts to an internal efficiency close to 100% when accounting for light-outcoupling losses.^13^ This demonstrates that the formed Ohmic hole contact prevents electrons from reaching the anode and subsequent nonradiative surface recombination, even in high forward bias. Insertion of a conventional electron-blocking layer with a low electron affinity, as typically used in OLEDs, did not improve the efficiency,^12^ proving that surface recombination at the anode is indeed completely suppressed, simply by the use of an Ohmic hole contact. As OLEDs typically operate above the built-in voltage where electrons drift toward the anode under the applied electric field, an increased built-in voltage cannot explain the absence of surface recombination in this OLED.

Although we agree that an increased built-in voltage reduces the electron density near the anode in a solar cell near short-circuit conditions, we would respectfully argue that band bending due to hole accumulation near the formed Ohmic contact would be the main contributor to the suppression of surface recombination of minority carriers. Near the maximum power point of a solar cell, the built-in voltage per se is of minor importance for minority-carrier surface recombination, as it is almost compensated by the applied voltage, whereas the formation of an Ohmic contact eliminates surface recombination even in high forward bias and promotes radiative bimolecular recombination instead. Additionally, the Ohmic contact minimizes energy—and thus voltage—losses upon the extraction of majority carriers.

Such Ohmic contacts can be universally achieved with an interlayer with a higher ionization energy than the used donor material. As the formation of Ohmic contacts invariably results in charge accumulation and band bending near the electrodes, surface recombination is effectively suppressed by using Ohmic contacts in organic devices.
